# Secretory granule neuroendocrine protein 1 (*SGNE1*) genetic variation and glucose intolerance in severe childhood and adult obesity

**DOI:** 10.1186/1471-2350-8-44

**Published:** 2007-07-07

**Authors:** Nabila Bouatia-Naji, Vincent Vatin, Cécile Lecoeur, Barbara Heude, Christine Proença, Jacques Veslot, Béatrice Jouret, Jean Tichet, Guillaume Charpentier, Michel Marre, Beverley Balkau, Philippe Froguel, David Meyre

**Affiliations:** 1 CNRS-8090-Institute of Biology, *Pasteur *Institute, Lille, France; 2 INSERM 780-IFR69, Univ *Paris Sud*, Villejuif, France; 3 INSERM U563, Children's Hospital, Toulouse, France; 4 IRSA, La Riche, France; 5 Endocrinology-Diabetology Unit, *Corbeil-Essonne *Hospital, France; 6 INSERM U695, *Bichat *Hospital, Paris, France; 7 Genomic Medicine, Hammersmith Hospital, Imperial College London, UK

## Abstract

**Background:**

7B2 is a regulator/activator of the prohormone convertase 2 which is involved in the processing of numerous neuropeptides, including insulin, glucagon and pro-opiomelanocortin. We have previously described a suggestive genetic linkage peak with childhood obesity on chr15q12-q14, where the 7B2 encoding gene, *SGNE1 *is located. The aim of this study is to analyze associations of *SGNE1 *genetic variation with obesity and metabolism related quantitative traits.

**Methods:**

We screened *SGNE1 *for genetic variants in obese children and genotyped 12 frequent single nucleotide polymorphisms (SNPs). Case control analyses were performed in 1,229 obese (534 children and 695 adults), 1,535 individuals with type 2 diabetes and 1,363 controls, all French Caucasians. We also studied 4,922 participants from the D.E.S.I.R prospective population-based cohort.

**Results:**

We did not find any association between *SGNE1 *SNPs and childhood or adult obesity. However, the 5' region SNP -1,701A>G associated with higher area under glucose curve after oral glucose tolerance test (p = 0.0005), higher HOMA-IR (p = 0.005) and lower insulinogenic index (p = 0.0003) in obese children. Similar trends were found in obese adults. SNP -1,701A>G did not associate with risk of T2D but tends to associate with incidence of type 2 diabetes (HR = 0.75 95%CI [0.55–1.01]; p = 0.06) in the prospective cohort.

**Conclusion:**

*SGNE1 *genetic variation does not contribute to obesity and common forms of T2D but may worsen glucose intolerance and insulin resistance, especially in the background of severe and early onset obesity. Further molecular studies are required to understand the molecular bases involved in this process.

## Background

Obesity is a major risk factor for metabolic disorders such as hypertension, dyslipidemia and type 2 diabetes (T2D) [1]. The increased prevalence of metabolic diseases is now affecting young populations as a direct consequence of the increase in childhood obesity [2]. There is evidence for a genetic contribution to the obesity epidemic, especially, for severe forms of obesity, including childhood obesity and severe adult obesity (BMI ≥ 40 Kg/m^2^) [3].

We previously performed a family based genome-wide scan using microsatellite markers and identified a suggestive obesity susceptibility linkage peak on chromosome 15q12-14 [4]. *SGNE1 *(Secretory Granule NeuroEndocrine protein 1) coding for 7B2 peptide is located within the 95% confidence interval of this linkage peak [4]. 7B2 mRNA and protein are located in either primarily neuronal (e.g hypothalamus) or endocrine (e.g pancreas and gastrointestinal tract) tissues [5]. 7B2 is a chaperone of the proprotein convertase (PC) 2 [6]. Pro7B2, the 7B2 peptide precursor, regulates the processing of proPC2 and is essential for the activation and regulation of PC2 in secretory granules of neuroendocrine cells [7].

PC2 is an endoprotease involved in the processing of several neuropeptides including proinsulin [8], proglucagon [9], pro-opiomelanocortin (POMC) [10] and the cocaine amphetamine regulated transcript (CART) [11]. 7B2 may also affect regulated hormone secretion [12]. Mice studies have shown that invalidation of the genes encoding either PC2 or 7B2 generates major hormone processing deficiency *in vivo *[13] and is lethal for gene encoding 7B2 in some genetic backgrounds in mice [14]. Interestingly, adrenalectomization of 7B2 knockout mice avoided the lethal phenotype but these animals developed severe obesity [15].

According to this physiological data and the genetic position of *SGNE1 *under an obesity linkage peak, we hypothesized that *SGNE1 *is a positional candidate gene for obesity and metabolic disorders in French Caucasians.

## Methods

### Subjects

#### Obese

We genotyped 630 obese children (defined as BMI > 97^th ^percentile for age and sex according to a French cohort [16]) selected from 424 nuclear families recruited in Lille through a national media campaign. Genotypes from 105 unrelated obese children recruited in the Children's hospital in Toulouse were also included and, in total, 735 obese children were studied. We selected 534 unrelated obese children for case/control studies. We also genotyped 1219 obese adults (BMI ≥ 30) recruited in Lille or at Hôtel-Dieu hospital in Paris. We selected 695 unrelated severely obese (BMI ≥ 40) and 620 unrelated moderately obese (30 ≤ BMI < 40) adults for case/control analyses. The moderate obese adults are sib pairs of severely obese and parents of obese children.

#### Type 2 diabetics

We genotyped 360 unrelated type 2 diabetic patients recruited by the *Centre National de la Recherche Scientifique *(CNRS) and *Institut Pasteur *in Lille and 1175 unrelated type 2 diabetic patients recruited from Endocrinology-Diabetology Department in the Corbeil-Essonne Hospital, Evry.

#### Controls

Control participants were composed of 623 individuals recruited by CNRS and Institut Pasteur in Lille or through the "*Fleurbaix-Laventie Ville Santé*" study. Additional 740 control subjects were selected among participants of the Data from the Epidemiology Study on the Insulin Resistance (D.E.S.I.R) study. Control participants were pooled for case control analyses. χ^2 ^tests did not show significant difference in genotype and allele frequencies between both control groups (0.253 ≤ p ≤ 0.886).

#### Cohort

Participants are from the D.E.S.I.R study, a 9 year follow-up study. We genotyped 4,922 subjects that had data available at baseline (M/F ratio: 49.6/50.4%; age = 47.2 ± 10.0 yrs; BMI = 24.7 ± 3.8 kg/m^2^). Among them, 4,387 subjects were normoglycemic (fasting plasma glucose<6.1 mmol/l) and 3,370 (77%) were followed for incident impaired fasting glucose (IFG) and T2D. A total of 3,645 participants were non obese (BMI<27 kgm^2^) at baseline, and 2,829 (78%) could be followed for overweight and obesity. After 9 years of follow-up, 2,053 subjects were both non obese and normoglycemic. T2D was defined as fasting plasma glucose ≥ 7.0 mmol/l and/or diabetes treatment.

All adult participants and parents of children signed informed consent. The genetic study was approved by ethical committees at corresponding recruitment centres in *Hôtel-Dieu *in Paris, C.H.R.U in Lille, *Bicêtre *Hospital for D.E.S.I.R study, Toulouse Children's Hospital.

### Measurements

Weight and height were measured by trained personnel and BMI was calculated as weight/height^2 ^(Kg/m^2^). During an oral glucose tolerance test (OGTT), participants received after a 12 h overnight fast 1 g glucose/kg if subject's weight was <50 kg or 75 g glucose if subject's weight was ≥ 50 kg. Blood samples were collected after 0, 30, 60, 90 and 120 min for measurement of plasma glucose using the glucose oxidase procedure and insulin using double-antibody radioimmunoassays. Glucose and insulin related traits were only analyzed in subgroups of 590 obese children and 575 obese adults, all normal glucose tolerant (NGT) (fasting glycemia<6.1 mmol/l). The insulinogenic index was calculated as (Ins_30min_-Ins_0min_/Glc_30min_-Glc_0min_) and HOMA-IR was calculated as Ins_0min_/[22.5e^-ln(Glc0min)^].

### Screening for SNPs

*SGNE1 *has 6 exons and spans 55 kb. We directly sequenced coding regions including exons, exons/introns boundaries, 3 kb upstream ATG codon (including non coding exon1 and intron1) and 1 Kb downstream the stop codon. We used DNA from 48 unrelated obese children and 24 unrelated control adults. PCR amplifications were purified with Montage PCR384 Multiscreen^®^S384PCR (Millipore). Sequencing was performed using the automated ABI Prism 3730xl DNA sequencer in combination with the Big Dye Terminator cycle (Applied Biosystems) and purification sequencing reaction with MultiScreen^®^SEQ384 filter plates (Millipore). Data from HapMap phase I was used to select TagSNPs (MAF≥0.05 in CEU population) to improve the genetic coverage of *SGNE1*.

### Genotyping

SNPs identified by direct sequencing were genotyped using either the LightCycler™ or LightTyper™ technologies (Roche). TagSNPs selected from HAPMAP I, and SNP -1,071A>G in the cohort were genotyped by the Applied Biosystems SNPlex™ technology based on the Oligonucleotide Ligation Assay (OLA) combined with multiplex PCR target amplification. Allelic discrimination was performed through capillary electrophoresis analysis using an Applied Biosystems 3730xl DNA Analyzer and GeneMapper3.7 software. All SNPs were re-genotyped in a subset of 379 individuals by direct sequencing to check for genotyping discrepancies. Genotyping error rates were ≤ 0.01 for all SNPs. Hardy-Weinberg equilibrium (HWE) was tested in controls and was verified for all SNPs (0.11 ≤ p ≤ 0.68).

### Statistical analyses

Tests for deviation from HWE and for association were performed by the De Finetti program [17]. Case control analyses for haplotype were performed using THESIAS software [18]. All possible combinations of 1 to 4 SNPs were tested. Haplotypes with a frequency of less than 1% were excluded. Analyses were conducted on inferred and observed genotypes. OGTT glycaemia and insulinemia were analysed by General Linear Model ANOVA for repeated measures. All other quantitative traits (QTs) were analyzed by a univariate ANOVA General Linear Model adjusted for sex, age, BMI, using SPSS 14.0 for windows. Haplotypes associations with QTs were performed using UNPHASED software [19]. Bonferroni correction was applied to quantitative traits as significance threshold (0.05) divided by the number of tests (12 SNPs × 9 QTs = 108) per population. Threshold of significance after adjustment for multiple testing was 0.0005. Survival curves were modelled and analysed by the Kaplan-Meier and Cox tests using R Foundation statistical software (version 2.4.0)

## Results

**Figure 1:**
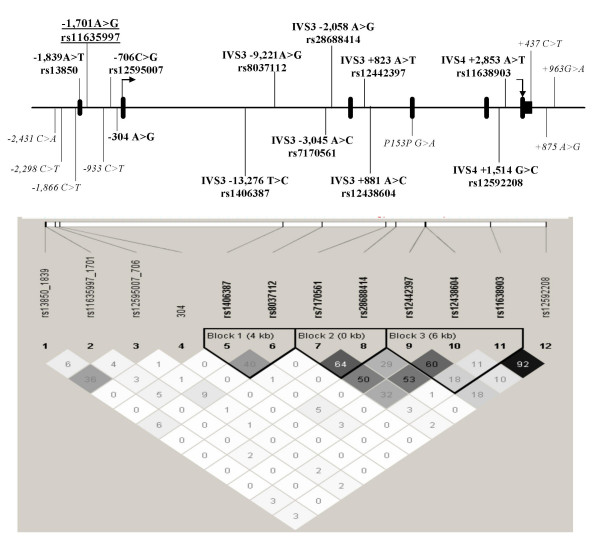
***SGNE1 *genetic variation (A) and LD (B) maps. Genetic variation map**: SNPs with MAF ≥ 0.05 are indicated in bold character. SNPs positions were allocated according to the human genome variation society nomenclature . IVS = intronic variation sequence. rs numbers were indicated for frequent SNPs when available. SNP 1,701A>G is underlined. **LD map**: LD map was generated using Haploview program. SNPs positions are on scale. LD (measured by correlation coefficient R^2^) was presented in boxes that correspond to the intersection of two SNPs (e.g. LD between rs13850_1839 and rs11635997_1071 is of 6%).

We identified four frequent (minor allele frequency ≥ 5%) and eight rare SNPs by direct sequencing. Frequent SNPs (-1,839A>T, -1,701A>G, -706C>G -304A>G) were all located in the 5' region. In order to improve the genetic coverage of *SGNE1*, we selected TagSNPs from the HapMap phase I. SNPs with a low genotype call rate (<70%) were not analyzed. Thus, eight TagSNPs all located in introns were added and 12 SNPs (Figure 1) were investigated for association with obesity and related phenotypes.

Clinical characteristics of the populations studied are presented in Table 1. Obesity case control analyses are presented in the Table 2. None of the *SGNE1 *SNPs or haplotype combination (data not shown) studied showed a significant association with severe forms of obesity in our populations. Thus, as expected, the contribution of these SNPs to linkage with childhood obesity observed on chromosome 15q12-q14 was not significant (data not shown). We also analyzed association of *SGNE1 *SNPs with moderate obesity (30 ≤ BMI ≤ 40). Case control analyses in additional 620 subjects did not show significant association with this less severe form of obesity (0.06 ≤ p ≤ 0.87; data not shown).

**Table 1:** Clinical characteristics of the populations studied.

	**Obese Children** **N = 735**	**Obese Adults** **N = 1315**	**T2D** **N = 1535**	**Control 1** **N = 623**	**Control 2** **N = 740**
**Age (years)**	11.3 ± 3.2	48 ± 13	59 ± 11	50.9 ± 12	53 ± 5.6
**BMI (kg/m**^2^**)**	28.8 ± 6.2	41.5 ± 8.8	29.3 ± 5.8	22.9 ± 2.3	23 ± 1.8
**Sex ratio (F/M)**	386/349	874/441	890/645	380/243	444/296
**Obesity (%)**	100	100	37	0	0
**Type 2 Diabetes (%)**	1	29	100	0	0
**NGT (%)**	94	52	0	100	100
**Fasting Glucose (mmol/l)**	4.95 ± 0.5	6.66 ± 2.6	9.40 ± 3.3	5.00 ± 0.4	5.05 ± 0.88
**Fasting Insulin (pmol/l)**	81.0 ± 58	88.2 ± 60	66.0 ± 54	33.5 ± 25	30.5 ± 22

**Table 2:** Obesity and T2D case control analyses for *SGNE1 *SNPs. OR: odds ratio. ORs and p values are for allele frequencies comparisons. χ^2 ^test and the p values were computed with De Finetti program [17]. All obese cases are the pooled sample of obese children and severely obese adults.

	**Genotype Frequencies**			**Allele Frequencies**		**OR [95%CI] (p value)**
	
**-1839 A>T rs13850**	AA	AT	TT	A	T	
**Obese Children**	384	128	21	0.84	0.16	1.10 [0.90–1.34] (0.335)
**Severely obese adults**	475	164	20	0.85	0.15	1.06 [0.88–1.28] (0.515)
**All obese**	859	292	41	0.84	0.16	1.08 [0.92–1.26] (0.327)
**Type 2 Diabetes**	777	306	20	0.84	0.16	1.08 [0.92–1.26] (0.339)
**Controls**	950	335	25	0.85	0.15	

**-1,701 A>G rs11635997**	AA	AG	GG	A	G	

**Obese Children**	191	267	90	0.59	0.41	1.14 [0.98–1.31] (0.078)
**Severely obese adults**	266	322	101	0.62	0.38	1.01 [0.88–1.16] (0.837)
**All obese**	457	589	191	0.61	0.39	1.07 [0.95–1.20] (0.255)
**Type 2 Diabetes**	554	722	208	0.62	0.38	1.03 [0.92–1.15] (0.619)
**Controls**	496	618	178	0.62	0.38	

**-706 C>G rs12595007**	CC	CG	GG	C	G	

**Obese Children**	384	111	10	0.87	0.13	0.83 [0.67–1.10] (0.081)
**Severely obese adults**	439	170	10	0.85	0.15	1.01 [0.84–1.21] (0.930)
**All obese**	823	281	20	0.86	0.14	0.92 [0.79–1.26] (0.343)
**Type 2 Diabetes**	976	352	21	0.85	0.15	0.95 [0.82–1.10] (0.511)
**Controls**	970	357	28	0.85	0.15	

**-304 A>G**	AA	AG	GG	A	G	

**Obese Children**	445	59	5	0.93	0.07	1.13 [0.84–1.51] (0.402)
**Severely obese adults**	587	91	2	0.93	0.07	1.17 [0.90–1.52] (0.240)
**All obese**	1032	150	7	0.93	0.07	1.15 [0.92–1.44] (0.210)
**Type 2 Diabetes**	1187	145	3	0.94	0.06	0.93 [0.74–1.17] (0.555)
**Controls**	1194	151	6	0.94	0.06	

**IVS3 -13,276 T>C rs1406387**	TT	TC	CC	T	C	

**Obese Children**	218	239	93	0.61	0.39	1.00 [0.86–1.15] (0.996)
**Severely obese adults**	243	340	102	0.60	0.40	1.05 [0.91–1.19] (0.510)
**All obese**	461	579	195	0.61	0.39	1.02 [0.92–1.14] (0.664)
**Type 2 Diabetes**	552	729	251	0.60	0.40	1.06 [0.96–1.18] (0.234)
**Controls**	499	666	191	0.61	0.39	

**IVS3-9,221 A>G rs8037112**	AA	AG	GG	A	G	

**Obese Children**	219	233	91	0.62	0.38	0.96 [0.83–1.11] (0.606)
**Severely obese adults**	260	322	95	0.62	0.38	0.95 [0.82–1.08] (0.422)
**All obese**	479	555	186	0.62	0.38	0.97 [0.85–1.07] (0.408)
**Type 2 Diabetes**	525	669	209	0.61	0.39	0.98 [0.88–1.10] (0.774)
**Controls**	481	666	190	0.61	0.39	

**IVS3 -3,045 A>C rs7170561**	AA	AC	CC	A	C	

**Obese Children**	248	211	66	0.67	0.33	1.04 [0.89–1.21] (0.621)
**Severely obese adults**	318	282	79	0.68	0.32	1.03 [0.89–1.18] (0.541)
**All obese**	566	493	145	0.67	0.33	1.03 [0.92–1.16] (0.598)
**Type 2 Diabetes**	627	603	155	0.67	0.33	1.05 [0.94–1.18] (0.372)
**Controls**	611	586	129	0.68	0.32	

**IVS3-2,058 A>Grs28688414**	AA	AG	GG	A	G	

**Obese Children**	307	184	33	0.76	0.24	1.00 [0.85–1.19] (0.959)
**Severely obese adults**	394	241	44	0.76	0.24	1.02 [0.88–1.19] (0.750)
**All obese**	701	425	77	0.76	0.24	1.01 [0.89–1.15] (0.809)
**Type 2 Diabetes**	803	480	94	0.76	0.24	1.03 [0.91–1.16] (0.678)
**Controls**	778	499	71	0.76	0.24	

**IVS3 +823 A>T rs12442397**	AA	AT	TT	A	T	

**Obese Children**	249	215	70	0.67	0.33	1.14 [0.98–1.32] (0.094)
**Severely obese adults**	320	293	64	0.69	0.31	1.03 [0.89–1.18] (0.670)
**All obese**	569	508	134	0.68	0.32	1.08 [0.96–0.1.21] (0.217)
**Type 2 Diabetes**	720	619	166	0.68	0.32	1.05 [0.94–1.18] (0.347)
**Controls**	642	583	117	0.70	0.30	

**IVS3 +881 A>C rs12438604**	AA	AC	CC	A	C	

**Obese Children**	323	184	35	0.77	0.23	1.16 [0.98–1.38] (0.073)
**Severely obese adults**	416	238	27	0.79	0.21	1.04 [0.89–1.22] (0.630)
**All obese**	739	422	62	0.78	0.22	1.09 [0.96–1.25] (0.181)
**Type 2 Diabetes**	903	463	81	0.78	0.22	1.05 [0.92–1.19] (0.459)
**Controls**	842	450	55	0.79	0.21	

**IVS4 +2,853 A>T rs11638903**	AA	AT	TT	A	T	

**Obese Children**	286	199	61	0.71	0.29	0.98 [0.84–1.15] (0.822)
**Severely obese adults**	335	291	56	0.70	0.30	0.99 [0.86–1.14] (0.885)
**All obese**	621	490	117	0.71	0.29	0.98 [0.87–1.11] (0.822)
**Type 2 Diabetes**	703	615	128	0.70	0.30	1.02 [0.91–1.14] (0.772)
**Controls**	657	588	109	0.70	0.30	

**IVS4 +1,514 G>C rs12592208**	GG	GC	CC	G	C	

**Obese Children**	281	195	65	0.70	0.30	0.96 [0.82–1.11] (0.591)
**Severely obese adults**	313	289	58	0.69	0.31	0.99 [0.85–1.14] (0.873)
**All obese**	594	484	123	0.70	0.30	0.97 [0.86–1.10] (0.678)
**Type 2 Diabetes**	716	647	139	0.69	0.31	0.99 [0.89–1.11] (0.911)
**Controls**	616	579	116	0.69	0.31	

**Figure 2:**
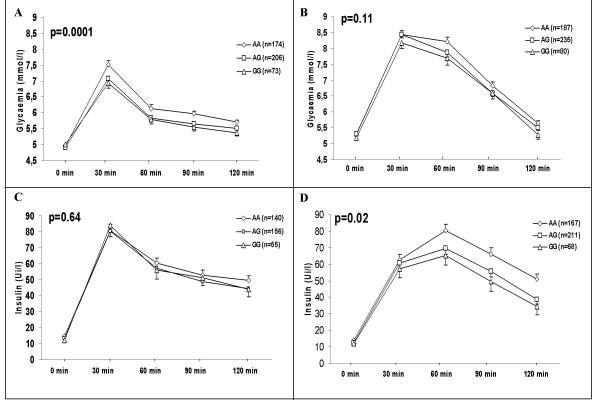
**Plasma glucose (A and B) and plasma insulin (C and D) after OGTTs in obese children (A and C) and obese adults (B and D) according to SNP -1,701A>G (rs11635997) genotypes**. Glucose and insulin levels during OGTT were compared using a general linear model ANOVA for repeated measures, adjusted for age, sex, and BMI. P values are for the dominant model (AA *vs*. AG+GG).

**Figure 3:**
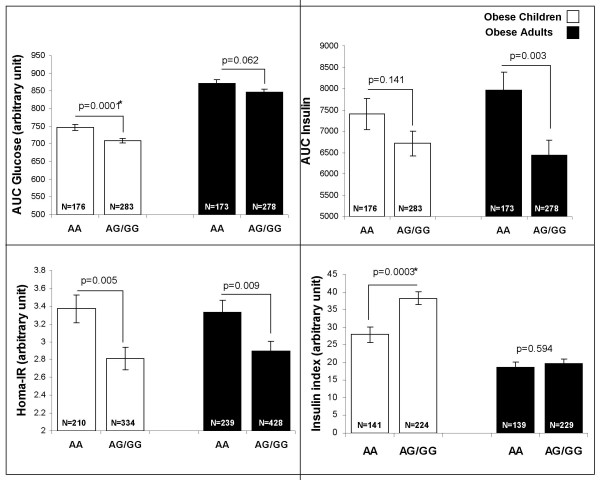
**SNP -1,701A>G associations with insulin and glucose related traits in obese children and obese adults**. Quantitative traits were compared using a General Linear Model ANOVA adjusted for age, sex, and BMI for a dominant model (AA *vs*. AG+GG).*: association is significant after Bonferroni correction (new threshold after correction = 0.0005, see statistical methods for more details).

We then investigated associations between *SGNE1 *SNPs and obesity related quantitative traits in obese subjects. SNP-1,701A>G, located in intron 1 which is part of the 5' region of *SGNE1 *(Figure 1), showed several strong and consistent associations with glucose intolerance. In obese children, despite no significant difference for fasting glucose, SNP -1,701A>G associated with an average 0.5 mmol/l increase (p = 0.0002) in glucose levels 30 min after an OGTT (Figure 2A) suggesting that -1,701A>G might alter the early insulin response after a glucose load. Indeed, the allele -1,701A carriers showed a 27% decrease in the insulinogenic index, an estimator of insulin secretion capacity (p = 0.0003) (Figure 3). Differences in glucose levels according to -1,701A>G genotypes were still significant after 120 min (p = 0.005). Thus, -1,701A>G associated with a 6% increase in the area under the curve (AUC) for glucose after OGTT (Figure 3; p = 0.0001). On the other hand, excepting for a borderline association with fasting insulin levels (p = 0.034), no significant differences were observed according to the -1,701A>G genotype for insulin levels during the OGTT (Figure 2B). Consequently, SNP -1,701A>G did not associate with AUC for insulin (p = 0.141) (Figure 3). However, the -1,701A allele associated with higher circulating fasting levels of pro-insulin in obese children (p = 0.037, data not shown).

In obese NGT adults, SNP -1,701A>G was also associated with glucose levels but only after 60 min (p = 0.038) and tended to be associated with higher levels at 90 min (p = 0.06) and 120 min (p = 0.085) (Figure 2C). Accordingly, we only observed a trend for an association between -1,701A>G and the AUC for glucose (p = 0.06) in obese adults (Figure 3). In addition to association with higher fasting insulin levels (p = 0.028), which was also seen in children, SNP -1,701A>G was associated with higher insulin levels after 60 min (p = 0.013), 90 min (p = 0.012) and 120 min (p = 0.001) (Figure 2D). Thus, SNP -1,701A>G associated with a higher AUC for insulin (p = 0.003) (Figure 3). SNP -1071A>G also associated with a higher insulin resistance index HOMA-IR in obese children (p = 0.005) and adults (p = 0.009) (Figure 3). The associations with AUC glucose and insulinogenic index continue reaching significance after Bonferroni correction in obese children only (for more details, see methods/statistical analyses). No haplotype combination showed stronger or independent association from -1,701A>G with glucose levels, insulinogenic index and HOMA-IR in obese children and obese adults (data not shown).

We then investigated the role of SNP -1,701A>G in the risk of T2D. We genotyped *SGNE1 *SNPs, including -1,701A>G in an independent population of 1,535 type 2 diabetics. *SGNE1 *SNPs or halpotypes (data not shown) did not associate with T2D (Table 2) in the whole population or in groups stratified for obesity or age of T2D onset before 45 y (data not shown). Logistic regression using age and sex as covariates did not show any significant association between T2D and SNP -1,701A>G either. Interestingly, SNP -1,701A>G was associated with a higher BMI in type 2 diabetic patients (mean AA = 33.6 ± 0.4 kg/m^2 ^*vs *AG/GG = 32.5 ± 0.3 kg/m^2^; p = 0.022, data not shown) but not HbA1C levels (mean AA = 8.26 ± 0.1 *vs*. AG/GG 8.42 ± 0.1; p = 0.29, data not shown).

**Figure 4:**
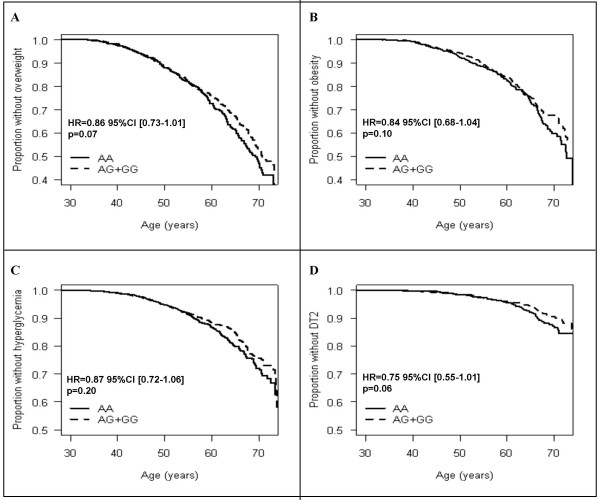
**Hyperglycemia incidence, by age at baseline, in the DESIR population according to SNP -1,701A>G genotype (dominant model)**. The genotype is considered a risk factor present since birth. The time scale is represented by age (continuous scale). The proportions with overweight (A), obesity (B), hyperglycemia (C) and T2D (D) were calculated within each genotype to assess the impact of SNP-1,701A>G on incidence of all phenotypes studied. Hazard ratios and p values indicated were obtained from Cox proportional hazard survival analyses with age, sex (overweight and obesity) and BMI (hyperglycaemia and T2D) as covariates among AA and AG+GG carriers during the 9 years of follow-up.

We then aimed to assess if the SNP -1,701A>G modulates traits related to obesity and glucose intolerance in a population-based cohort. Thus, SNP-1,701A>G was also genotyped in 4,922 participants from the D.E.S.I.R prospective study. Cross sectional analyses at baseline did not show an association of SNP -1,701A>G with fasting glucose, fasting insulin, HbA1c levels, HOMA-IR or BMI (data not shown). Only fasting glucose and fasting insulin levels were available for the cohort participants. After 9 years follow-up, allele -1,701G carriers tend to be at lower risk of incidence of overweight (HR = 0.86; p = 0.07), obesity (HR = 0.84; p = 0.10) and T2D (HR = 0.75; p = 0.06) (Figure 4).

## Discussion

Here we describe the genetic study of the pituitary peptide 7B2 encoding gene *SGNE1*, candidate gene for obesity and related metabolic traits. Our study does not provide evidence of association between *SGNE1 *genetic variation and severe forms of obesity in large populations of French Caucasians, including children and adults. However, one SNP, the -1,701A>G, located in the 5' region of *SGNE1*, associate with impaired glucose tolerance in the context of severe obesity but does not increase the risk for T2D. A limitation of our study design is that SNP tagging was based on data from HapMap phase I. According to HapMap phase II data [20] genotypes from 18 additional TagSNPs would be necessary for an extensive genetic coverage and comprehensive association analyses of *SGNE1 *with metabolic diseases.

Our results support that SNP -1,071A>G, although not associated with obesity, may contribute to higher glucose levels after OGTT. Similar findings were previously reported for *USF1 *(*Upstream Stimulatory Factor 1*), where SNPs were associated with higher glucose after OGTT, although allelic frequencies were similar between T2D cases and controls [21]. SNP -1,701A>G also associated with higher HOMA-IR and lower insulinogenic index in obese children. As far as adults are concerned, -1,701A allele carriers compensate the decreased insulin secretion capacity over time but their insulin resistance remained impaired. A high proportion (~75%) of our obese adults self-reported overweight since childhood which may explain a shared genetic background of both populations as previously reported [22]. However, their metabolic profile is fairly diverse: 94% of obese children are glucose tolerant after OGTT, against only 52% of the severely obese adults. These data may elucidate the observed heterogeneity between quantitative traits associations in children and adults. We also believe that obesity related quantitative traits results should be interpreted with caution in adults as these analyses are more meaningful in obese children [23].

SNP-1,701A>G is located in intron 1 and is part of the 5' region of *SGNE1*. It has been shown that intron 1 sequences are essential for transcriptional activity of *SGNE1 *in human cell lines [24]. A recent study has also shown that intron 1 of *SGNE1 *is hypermethylated and epigenetically silenced in medulloblastomas [25]. This data supports an important role of intron 1 of *SGNE1 *and a potential functional role of SNP -1,701A>G in the regulation of *SGNE1*. According to *in silico *analyses [26], SNP -1,701A creates a theoretical binding site for *TCF/LEF1 *(Transcription Factor T-Cell specific/Lymphoid Enhancer binding Factor1). To our knowledge, *TCF/LEF1 *role in metabolism has not been described. We note that *TCF/LEF1 *belongs to the Wnt signaling pathway that includes *TCL7L2*, which harbours highly associated SNPs with T2D [27]. Further molecular studies are required to elucidate how SNP -1,701A>G would affect the expression and function of *SGNE1 *and ultimately impaired metabolism. An increase of 7B2 has been reported in pancreas from *ob/ob *mice compared to wild type mice [28] suggesting that 7B2 may increase in insulin resistance state. Thus, as 7B2 is immunologically detected in human plasma [29], analyses of association between SNP -1,701A>G and 7B2 plasma levels in our populations would provide a value indication about how this genetic variant might impact *SGNE1 *functionality and consequently, glucose tolerance and insulin resistance.

As a protein helper and activator of PC2, 7B2 is involved in the maturation of a large spectrum of molecules playing central roles in weight and glucose homeostasis [5]. 7B2 and PC2 are highly expressed in secretory granules of pancreatic islets and are involved in processing of insulin in β-cells and glucagon in α-cell [5]. High levels of circulating pro-insulin and pro-glucagon have been described in 7B2 knock-out mice confirming the role of 7B2 in insulin and glucagon maturation [30]. If associations and functionality of SNP -1,701A>G are confirmed, we hypothesize that higher glucose levels and lower insulin secretion observed in -1,701A allele carriers could be direct, as a consequence of impairment of the processing and/or secretion of insulin. The observed SNP -1,701A>G associations with higher pro-insulin levels and lower insulinogenic index in obese children support this hypothesis. Insulin secretion could also be indirectly affected through alteration of glucagon processing that requires both PC2 and 7B2. Thus, impairment of glucagon processing may affect insulin secretion and results in increase glucose levels. This hypothesis is supported by a recent study where low circulating glucagon levels accompanied with low 7B2 mRNA and protein levels (but not PC2 levels) were reported in pancreas of a mouse strain highly predisposed to age associated glucose intolerance [31]. Because of the pleitropic function of 7B2, several mechanisms would result from the alteration of its regulation. Other metabolic pathways could also be affected, such as the processing of cholecystokinin (CCK) [32], a gastrointestine peptide that stimulates insulin secretion from β-cells in healthy subjects [33] and the maturation of anorexigenic neuropeptides CART [11] and POMC [10] involved in the regulation of energy balance.

## Conclusion

In summary, we report genetic evidence for the role of *SGNE1 *genetic variation in glucose intolerance and insulin resistance in the background of young onset obesity. Further analyses are required to replicate our findings and to understand the molecular bases of *SGNE1 *role in the genetic susceptibility to the risk of glucose intolerance in response to glucose load.

## Abbreviations

AUC Area under curve

BMI Body mass index

CART Cocaine amphetamine regulated transcript

CCK Cholecystokinin

HOMA-IR Homeostasis model assessment – insulin resistance

IFG Intolerant fasting glucose

NGT Normal glucose tolerant

OGTT Oral glucose tolerance test

PC2 Proprotein convertase 2

PCR Pomymerase chain reaction

POMC Pro-opiomelanocortin

SGNE1 Secretory granule neuroendocrine protein 1

SNP Single nucleotide polymorphism

T2D Type 2 diabetes

TCF/LEF1 Transcription factor t-cell specific/lymphoid enhancer binding factor 1

TCF7L2 Transcription factor t-cell specific 7-like 2

USF1 Upstream Stimulatory Factor 1

## Competing interests

The author(s) declare that they have no competing interests.

## Authors' contributions

NBN and VV performed the genotyping. NBN, CL, JV, CP and DM performed the statistical analyses. DNA was provided by GC, BH, JT, MM and BB. NBN drafted the manuscript under PF and DM supervision. DM and PF supervised the study. All authors read and approved the final manuscript.

## Pre-publication history

The pre-publication history for this paper can be accessed here:


